# Gender Differences in the Effects of Exercise Interventions on Alzheimer’s Disease

**DOI:** 10.3390/brainsci15080812

**Published:** 2025-07-28

**Authors:** Yahong Dong, Lei Shi, Yixiao Ma, Tong Liu, Yingjie Sun, Qiguan Jin

**Affiliations:** College of Physical Education, Yangzhou University, Yangzhou 225000, China; dongyahong@139.com (Y.D.); dx120240095@stu.yzu.edu.cn (L.S.); myixiao@163.com (Y.M.); liut9904@163.com (T.L.); sunyingj@outlook.com (Y.S.)

**Keywords:** Alzheimer’s disease, gender differences, exercise intervention, cognitive function

## Abstract

Alzheimer’s disease (AD) is a progressive neurodegenerative disorder primarily characterized by memory loss, cognitive decline, and structural brain atrophy. Substantial sex differences have been observed in its incidence, clinical trajectory, and response to treatment. Women are disproportionately affected, exhibiting faster progression and more severe cognitive impairment. Exercise has emerged as a promising non-pharmacological intervention to mitigate AD-related decline, yet growing evidence reveals that its benefits vary by sex. This review synthesizes current findings from human and animal studies, focusing on how exercise impacts AD differently in males and females. In women, exercise is more strongly associated with improvements in cognitive function, neurotrophic support, and emotional regulation. In men, benefits tend to involve structural preservation and oxidative adaptations. Underlying mechanisms include differential hormonal profiles, inflammatory responses, and neuroplastic signaling pathways. These findings underscore the need to consider sex as a biological variable in AD research. Developing sex-specific exercise strategies may enhance therapeutic outcomes and support more individualized approaches in AD prevention and care.

## 1. Introduction

Alzheimer’s disease (AD), the most common form of dementia, accounts for approximately 60–80% of all dementia cases. Amidst the accelerating global aging trend, the incidence of AD continues to rise, a major and urgent public health challenge requiring prompt intervention [1]. The pathological hallmarks of AD include the accumulation of amyloid-beta (Aβ) plaques in the cerebral cortex and hippocampus, the formation of neurofibrillary tangles (NFT), and progressive neurodegeneration. These processes compromise key metabolic pathways, trigger neuronal apoptosis, and result in extensive neuronal loss, ultimately facilitating the clinical transition from mild cognitive impairment (MCI) to overt AD. Furthermore, AD is frequently associated with cerebral atrophy, primarily due to extensive neuronal loss and impaired cerebral glucose metabolism. Clinically, it manifests as marked deficits in memory, language, reasoning, and executive functioning, significantly impairing patients’ daily life and social independence [2].

Gender is considered an important associated factor in AD. Research indicates that females represent approximately 65–70% of the total AD population [3]. Recent research results show that although women account for a larger proportion of AD cases, there is no significant difference in the overall rate of cognitive decline between genders. However, compared with men, women diagnosed with mild AD may experience faster disease progression in the early stage, highlighting the stage potential of gender differences in clinical outcomes [4]. This phenomenon is not only linked to women’s relatively longer lifespans but also involves biological aspects such as postmenopausal declines in estrogen levels and a faster rate of hippocampal atrophy. The decrease in estrogen levels reduces receptor sensitivity. It weakens synaptic signal transmission, thereby aggravating the deposition of β-amyloid and the formation of neurofibrillary tangles, which facilitate the progression of AD pathology. At the same time, fluctuations in estrogen levels reduce the body’s antioxidant and anti-inflammatory capacity, making memory-related regions such as the hippocampus more prone to degenerative atrophy [5]. Moreover, in terms of economic impact and caregiving demands, women with AD often require extended periods of support. Neuropsychiatric symptoms (NPS), including depression and anxiety, tend to be more severe, placing an additional strain on social and economic resources [6,7].

Despite the availability of several pharmacological interventions for AD, their overall efficacy in altering disease progression remains limited. Cholinesterase inhibitors such as donepezil have demonstrated only modest benefits on cognition, activities of daily living, and global clinical assessments over 12 to 24 weeks in patients with mild to severe AD, with higher doses (23 mg/day) associated with increased rates of adverse events and treatment discontinuation [8]. Similarly, the N-methyl-D-aspartate receptor antagonist memantine modestly slows clinical deterioration in moderate-to-severe AD, but does not significantly alleviate neuropsychiatric symptoms, and remains linked to increased caregiver burden [9]. More recent agents, including anti-amyloid monoclonal antibodies such as lecanemab, have shown moderate reductions in cognitive and functional decline in early-stage AD over an 18-month period. However, these effects are coupled with notable adverse events and substantial uncertainty regarding long-term safety and effectiveness [10]. Network meta-analyses have confirmed slight cognitive and functional benefits across pharmacological classes but consistently highlight a lack of efficacy in improving neuropsychiatric symptoms, with drug choice largely depending on disease severity [11]. Furthermore, cost-effectiveness analyses have raised serious concerns—most notably with aducanumab, which demonstrated only minimal health gains at an annual cost of $56,000, resulting in incremental cost-effectiveness ratios far exceeding acceptable thresholds, even under optimistic scenarios [12]. Collectively, these limitations underscore the urgent need for safe, accessible, and cost-effective non-pharmacological strategies.

Exercise has been widely recognized as a practical non-pharmacological approach for improving cognitive performance, maintaining brain structural integrity, and supporting functional independence in individuals with AD [13]. Regular physical activity not only contributes to the reduction of AD-related risk factors but also confers neuroprotective effects by enhancing the production of neurotrophic factors, mitigating inflammatory responses, and restoring redox homeostasis [14]. Notably, different types of exercise may exert distinct physiological effects on the brain. Aerobic exercise, characterized by rhythmic, continuous movements of large muscle groups, has shown moderate evidence for alleviating neuropsychiatric symptoms and improving quality of life in individuals with AD [15]. In contrast, resistance exercise, which applies external loads to induce skeletal muscle hypertrophy and strength gains, elicits different neuroprotective responses. Resistance exercise is associated with increased cortical and hippocampal volume, enhanced neuroplasticity, and cognitive improvements [16]. Mechanistically, aerobic exercise is primarily linked to the regulation of hippocampal genes such as APP, APOE, and MAPT, as well as elevated levels of brain-derived neurotrophic factor (BDNF). Conversely, resistance exercise predominantly upregulates insulin-like growth factor 1 (IGF-1) and its receptor (IGF-1R), modulating the AKT1 signaling pathway [17]. Despite their distinct mechanisms, both aerobic exercise and resistance exercise contribute to improved neurocognitive performance, including higher task accuracy, faster reaction times, and enhanced P3 amplitude in event-related potentials. Furthermore, aerobic exercise significantly increases peripheral BDNF and reduces insulin, TNF-α, and IL-15 levels, while resistance exercise elevates IGF-1 and reduces IL-15 [18]. These results suggest that both aerobic exercise and resistance exercise effectively promote neurotrophic signaling and attenuate inflammatory cytokines, yet through divergent molecular pathways, ultimately leading to convergent cognitive benefits.

However, current research has given limited attention to how gender differences influence the outcomes of exercise interventions, leaving our understanding of the differential benefits for male and female AD patients incomplete. This article explores AD from a gender perspective, examining its pathological background, the potential impact of exercise on AD, and the differential effects of exercise interventions.

## 2. Gender Differences in AD Formation

### 2.1. Epidemiological Differences

AD presents a growing global public health challenge with significant societal and economic burdens. In the United States, someone develops AD every 69 s, a rate projected to double by 2050. The total direct cost of care for AD and other dementias was estimated at $183 billion in 2010 and is expected to reach $1.1 trillion by 2050 [19]. In 2024 alone, an estimated 6.9 million Americans aged 65 and older are living with Alzheimer’s dementia, with 74% of them aged 75 or older. The prevalence of AD dementia increases substantially with age: approximately 5.0% among those aged 65–74, 13.2% in those aged 75–84, and 33.4% in those aged 85 and above. From 2000 to 2021, AD-related mortality rates rose by 41% in people aged 65–74, 54% in those 75–84, and 86% in those aged 85 and older, making AD a leading cause of death among the elderly [20].

Globally, AD demonstrates a pronounced gender disparity, with a higher prevalence among women. Epidemiological data indicate that of the approximately 32 million individuals affected by AD worldwide, about 65% are women, and this difference becomes more evident with advancing age [21]. In the United States, as of 2020, 3.74 million women aged 65 and older were diagnosed with AD, compared to 2.34 million men, highlighting the gender gap in disease burden [22]. Similarly, data from China in 2022 revealed that the prevalence of AD among individuals aged 75 and above was 5.98%, with a higher prevalence in females (4.93%) compared to males (3.09%), further highlighting the challenges posed by gender differences in AD [23]. According to a 20-year follow-up from the Framingham study involving 2611 cognitively healthy individuals, 198 participants developed dementia, including 120 cases of AD. Among those aged 65 and older, the lifetime risk of developing AD or other dementias was 12% for women—nearly twice that of men (6.3%). Additional analysis from the same study indicated that between the ages of 65 and 100, the cumulative incidence of AD reached 28.1% in women, surpassing the 25.5% observed in men [24]. While longer life expectancy in women partially explains this difference, it does not fully account for the broader and more persistent gender disparity in AD risk and incidence [25]. A key contributing factor is the fluctuation in estrogen levels. Estrogen serves as a critical neuromodulator in women, playing an essential role in maintaining cognitive function, synaptic plasticity, and neuronal survival. Its neuroprotective effects include the regulation of amyloid precursor protein processing and the support of BDNF signaling—both of which are vital to the pathogenesis of Alzheimer’s disease [26].

### 2.2. Phenotypic Differences

The amyloid hypothesis remains central to understanding the pathophysiology of AD, suggesting that abnormal accumulation of Aβ, derived from amyloid precursor protein, leads to neuronal toxicity in the central nervous system. Recent studies have also linked Aβ formation with impaired autophagy, reduced cerebral blood flow, altered ACHE activity, and changes in LRP1 expression, all of which contribute to disease progression [27]. Recently, increasing attention has been given to the role of gender in influencing these pathological processes. Gender differences in AD are reflected in both neuropathological characteristics and clinical manifestations. Evidence from diagnostic assessments, neuroimaging, cerebrospinal fluid analysis, and autopsy studies consistently indicates that women exhibit more complex and severe pathological and symptomatic profiles than men (Table 1 and Table 2) [28,29].

At the pathological level, women tend to experience a later onset of symptoms, a longer disease duration, and faster progression. Female patients often present with smaller brain volumes and more rapid advancement through Braak stages, potentially contributing to accelerated cognitive deterioration. Compared to men, women show slightly higher levels of Aβ accumulation in the hippocampus and increased NFT density [30]. Autopsy studies reveal that such pathological changes in women typically emerge during late middle age. In the early phases of Aβ and NFT deposition, women demonstrate more prominent alterations, and those carrying the apolipoprotein E4 (APOE4) allele are especially prone to rapid NFT progression [31]. Building on these findings, a recent large-scale meta-analysis of longitudinal PET data revealed that women, particularly in the presence of elevated Aβ, accumulate tau more rapidly than age-matched men. This sex difference in tau progression is most pronounced in the temporal and occipital lobes, and appears to be further influenced by APOEε4 status [32]. Complementary evidence from biomarker studies supports the notion of increased tau pathology in women. Women are more likely to exhibit elevated total tau (T-tau) and phosphorylated tau (p-tau) levels in both cerebrospinal fluid and plasma, suggesting heightened vulnerability to neurodegeneration [33]. In animal models, female Tg2576 mice develop Aβ plaques that are nearly three times larger than those in males, with significantly higher levels of Aβ40 and Aβ42 in the brain, emphasizing a heavier pathological burden in females. This difference may be closely related to hormonal, genetic, and neurobiological factors [34].

At the clinical level, recent reviews have highlighted significant gender differences in the presentation of AD, particularly during the preclinical and mild cognitive impairment (MCI) stages. Women’s relative advantage in verbal memory may mask early cognitive deficits, thereby delaying diagnosis and reducing opportunities for timely intervention. This phenomenon is thought to align with the cognitive reserve theory, which posits that women may demonstrate domain-specific resilience in certain cognitive functions. However, once symptoms become apparent, women often experience a more rapid decline in cognitive function than men [35]. Empirical studies have further demonstrated that female AD patients exhibit a higher incidence and a faster rate of cognitive decline compared to males [36]. Irvine et al. further reported that females perform worse than males in visuospatial ability, language comprehension, and semantic memory tasks, indicating broader cognitive vulnerability in these domains [37]. Structural imaging studies have identified hippocampal atrophy and asymmetry as key markers of disease progression. Interestingly, men exhibit greater hippocampal asymmetry than women during normal aging and early disease stages, with asymmetry differences reaching up to 2.04% [38]. However, despite this structural advantage, women experience more pronounced hippocampal atrophy as the disease progresses. Holland et al. reported that at baseline, there were no significant interactions between gender, Aβ42, or total tau and bilateral hippocampal volume, executive function, or memory performance. However, during longitudinal data, women with lower cerebrospinal fluid Aβ42 levels showed greater left hippocampal atrophy and a more rapid decline in memory and executive function. Additionally, when total tau levels were elevated, women exhibited a stronger association between left hippocampal atrophy and deterioration in executive performance [39]. Notably, despite deficits in other cognitive domains, women often retain relative strengths in verbal memory during early stages, which may obscure clinical signs of MCI, leading to delayed diagnosis and more advanced disease stages at the time of detection [40]. In terms of subjective cognitive decline or memory complaints (SCD or SMC), women report higher frequencies than men, especially in conjunction with depression or dementia, where symptoms are more pronounced [33,41]. Additionally, women with AD are more likely to experience neuropsychiatric symptoms such as depression, anxiety, and delusions. In contrast, male patients more frequently present with apathy or emotional indifference [42]. Postmortem studies further support these clinical observations, revealing a stronger association between AD pathology and cognitive symptoms in women than in men [43]. Collectively, these findings suggest that women bear a disproportionately greater disease burden throughout the course of AD.

### 2.3. Contributing Factors to Gender Differences in AD Formation

#### 2.3.1. Biological Factors

Gender disparities in AD cannot be solely attributed to differences in life expectancy. A range of biological factors—including hormonal fluctuations, immune responses, genetic predispositions, and brain structural characteristics—also contribute significantly to the observed differences in disease risk and progression [44].

Hormonal variation is one of the key biological mechanisms contributing to gender differences in AD [45]. Sex-specific influences, largely driven by steroid hormones, shape brain structure and function throughout the lifespan, playing a vital role in both brain health and the development of neurodegenerative diseases. These hormones exert gender-specific effects on neuroplasticity, particularly within the adult hippocampus—a region highly susceptible to AD-related damage [46]. Among these hormones, estrogen plays a neuroprotective role in the female brain by regulating brain-derived neurotrophic factor (BDNF) and APOE expression, maintaining neuronal integrity, and enhancing synaptic plasticity and cognitive function while mitigating apoptosis and inflammatory responses [26,47]. However, estrogen levels drop significantly during perimenopause and postmenopause, impairing the clearance of Aβ and phosphorylated tau [48]. Scheyer et al. found that women during this period often show decreased brain glucose metabolism and cognitive decline, closely related to estrogen fluctuations [49]. Hormone replacement therapy (HRT) can partially delay these changes, further supporting the critical role of estrogen in AD pathogenesis [50]. Similarly, declining testosterone levels in men are linked to increased AD risk [51]. At the molecular level, testosterone has been shown to reduce neuronal secretion of Aβ peptides by promoting non-amyloidogenic processing of amyloid precursor protein through increased sβAPPα production [52]. Clinically, lower levels of free testosterone correlate with elevated luteinizing hormone, follicle-stimulating hormone, and sex hormone-binding globulin, all of which are commonly observed in male AD patients. Notably, low free testosterone has been identified as an independent predictor of AD, and its reduction is significantly associated with cognitive decline and metabolic dysregulation [53]. Testosterone supplementation has demonstrated selective cognitive benefits, particularly in improving visuospatial memory in hypogonadal men with mild-to-moderate AD or mild cognitive impairment [54,55]. Furthermore, large-scale longitudinal data confirm that lower total testosterone and higher sex hormone-binding globulin are independently associated with increased AD incidence in aging me [56]. However, because testosterone declines more gradually with age, cognitive deterioration in men tends to progress more slowly, potentially lowering their mid- and late-life vulnerability to AD [57].

Gender-based differences in immune responses further contribute to gender-specific AD pathology [58]. Casaletto et al. reported that microglial activation in response to amyloid-β was more robust in females than in males, and significantly mediated the effect of Aβ on tau pathology in women but not in men. Their mediation analysis showed that in females, microglial activation accounted for 57% of the total effect of Aβ on tau, suggesting a stronger immunopathological linkage between amyloid and tau in women with Alzheimer’s disease [59]. While moderate immune responses facilitate the clearance of toxic proteins, sustained activation of microglia in females leads to excessive release of pro-inflammatory cytokines and reactive oxygen species, inducing oxidative stress and accelerating neuronal damage [60,61]. Recent studies have shown that women with Alzheimer’s disease exhibit a robust Aβ-plaque-independent microglial response, which shows stronger associations with tau pathology than observed in males. This sex-divergent mechanism underscores the imperative to integrate gender perspectives in AD neuroinflammation research [62]. In APP/PS1 mouse models, this inflammatory profile corresponds to a glycolytic, less phagocytic microglial phenotype that promotes Aβ deposition [63]. Moreover, female microglia show increased iron accumulation and mitochondrial dysfunction, contributing to heightened oxidative damage and neurodegeneration [64].In a word, these sex-specific features of microglial activation and metabolic dysregulation help explain the higher susceptibility and severity of AD in females [65]. Recent transcriptomic analyses have identified SERPINA3, a serine protease inhibitor associated with neuroinflammation, as a key modulator of sex-specific immune responses in AD. SERPINA3 expression is significantly elevated in male AD patients compared to non-demented controls, with distinct sex differences observed in healthy individuals where females display higher SERPINA3 levels across multiple brain regions. Importantly, SERPINA3 levels in males strongly distinguish AD patients from controls, suggesting its potential as a sex-specific biomarker. SERPINA3 expression correlates positively with clinical dementia severity and neurofibrillary tangle burden, and shows strong association with microglial and astrocytic markers, indicating a central role in modulating neuroinflammatory processes in AD. The interaction between SERPINA3 and CHI3L1 proteins further supports its involvement in disease mechanisms. These findings underscore the complexity of neuroinflammation in AD and highlight the necessity to consider sex differences in inflammatory pathways for therapeutic development [66].

The APOE4 allele, the strongest genetic risk factor for late-onset AD, exerts gender-specific effects [67,68]. Female APOE4 carriers exhibit more severe hippocampal atrophy, cortical thinning, and metabolic abnormalities than their male counterparts [69]. These differences may be amplified by physiological changes during perimenopause and menopause, thereby exacerbating the harmful effects of APOE4 [49]. Recent evidence indicates that plasma BDNF partially mediates sex differences in verbal learning and memory. Women generally show higher BDNF levels and superior verbal learning and memory performance compared to men, especially among APOE4 non-carriers. Notably, the protective effect of BDNF on language memory is attenuated in female APOE4 carriers, although their verbal learning and memory scores remain higher than those of males. This discovery reveals an interaction between gender and APOE4, jointly influencing the regulatory mechanism of BDNF on cognitive function, providing a new perspective for understanding gender differences in AD risk [70].

Structural and functional brain differences also exacerbate gender differences in AD. Ritchie et al. found that female brains tend to atrophy more rapidly during cognitive and neurodegenerative decline [71]. Structurally, male brains typically have larger volume, surface area, and white matter fraction, while females show greater cortical thickness and more intricate white matter connectivity. Functionally, male brains display stronger intra-hemispheric connectivity, facilitating sensory-motor integration, whereas female brains favor inter-hemispheric connectivity, supporting analytic and intuitive processing. These structural and functional distinctions may lead to gender-specific patterns of adaptation and response when faced with AD-related pathological changes [72].

Disruptions in protein homeostasis also contribute to gender disparities in AD, particularly in Aβ deposition and tau hyperphosphorylation [73]. Women tend to show elevated tau pathology with higher Aβ burden, suggesting a lower adaptive capacity to tau pathology [74]. Additionally, while women have higher brain glucose metabolism and cerebral blood flow during reproductive age, these metabolic advantages diminish with aging and declining estrogen, transforming into risk factors for AD [75]. These findings underscore the unique pathological trajectories in women and emphasize the importance of gender-specific mechanisms in AD.

**Table 1:** Summary of gender differences in Alzheimer’s disease.

Category	Female	Male	Reference
Age of Onset and Disease Course	1. Higher AD prevalence with increasing age. 2. Greater disease burden among older death age groups.	1. Earlier cognitive symptom onset. 2. Shorter disease duration. 3. More atypical (non-amnestic) presentations.	[30]
Pathological Accumulation	1. Greater brain region involvement from late middle age. 2. Extensive accumulation of SP was detected at NFT stages I, II, and III. (Specifically observed in females carrying the APOE4 allele).	1. Extensive accumulation of SP was detected at NFTs stages IV, V, and VI.	[31]
Tau accumulation in high Aβ or APOEε4	1. Faster tau accumulation in inferior temporal, temporal fusiform, and lateral occipital regions. 2. In APOEε4 carriers (inferior temporal).	1. Slower tau accumulation in these regions.	[32]
Neurodegeneration Biomarkers	1. Higher brain glucose metabolism. 2. Greater cortical thickness. 3. Elevated CSF T-tau levels.	1. Higher lifelong levels of neurofilament light chain (NfL).	[33]
Aβ burden (Animal Model)	1. Female mice exhibit significantly greater senile plaque burden. 2. Higher brain levels of Aβ40 and Aβ42 compared to male mice.	N/A	[34]
Cognitive Decline Progression	1. Greater decline in ADAS-Cog11 scores in both APOE ε4 carriers and non-carriers. 2. Faster cognitive decline with lower CSF Aβ42 levels. 3. More pronounced cognitive deterioration during the MCI stage; smaller baseline hippocampal volume but larger after normalization. 4. Greater cognitive decline in APOEε4 positive MCI subjects.	1. Smaller decline in ADAS-Cog11 scores in both APOEε4 carriers and non-carriers compared to females. 2. Less cognitive deterioration during MCI stage; larger baseline hippocampal volume but smaller after intracranial volume normalization. 3. Slower cognitive decline in APOEε4 positive MCI subjects.	[36]
Clinical Cognitive Performance	1. Worse performance in visuospatial, language, and semantic memory tasks.	1. Advantages in visuospatial, linguistic, and semantic memory tasks.	[37]
Hippocampal Asymmetry	1. Lower hippocampal volumetric asymmetry.	1. Higher hippocampal volumetric asymmetry.	[38]
Hippocampal Atrophy Based on Biomarker Status	1. More pronounced left hippocampal atrophy with a decrease in Aβ42 in CSF 2. Memory and executive function decline faster. 3. Tau is elevated in CSF, and left hippocampus is atrophied.	N/A	[39]
Temporal Lobe Glucose Metabolic Rate and Verbal Memory	1. Higher Temporal Lobe Glucose Metabolic Rate (TLGluMR) is associated with better verbal memory performance. 2. The female advantage in verbal memory is most pronounced at moderate to high TLGluMR levels.	N/A	[40]
Subjective Cognitive Decline/ Self-Memory Complaint (SCD/SMC)	1. SMC is significantly associated with increased dementia risk across all risk periods, including long-term follow-up. 2. After adjustment for education, marital status, depressive symptoms, and global cognition, SMC independently predicts dementia risk, whereas IADL limitations are not associated.	1. IADL limitations are associated with an increased risk of dementia, limited to the first 5 years. 2. After adjustment for education, marital status, depressive symptoms, and global cognition, only IADL limitations remain significantly associated with dementia risk, while SMC is not predictive.	[41]
Neuropsychiatric Symptoms (NPS)	1. Greater NPS burden. 2. More frequent depression, anxiety, and delusions.	1. It is often associated with indifference.	[42]
Clinicopathological Correlation	1. Stronger correlation between AD pathology and clinical symptoms.	1. Weaker correlation between AD pathology and clinical symptoms.	[43]

(All studies cited include comparative data for both male and female participants. Instances marked as ‘N/A’ do not indicate missing data but reflect either non-significant findings or a lesser degree of change in males relative to females.)

**Table 2:** Quantitative comparisons of gender-specific biomarkers and clinical features in AD pathology.

Indicator	Female	Male	Reference
Age distribution curve of AD	U-shape (intersecting at age 70)	Inverted U-shape	[30]
AD subtype	Limbic predominant subtype	Hippocampus-sparing subtype	[30]
Regional distribution of hippocampal NFT counts	Gradual increase	Gradual decrease	[30]
Regional distribution of neocortical NFT counts	Gradual decrease	Gradual decrease	[30]
Braak NFTs stage	Higher	N/A	[30]
Amyloid plaque burden	Slightly higher	N/A	[30]
Hippocampal asymmetry value	3.46%	5.5%	[38]
Number of individuals meeting probable AD clinical criteria	34 individuals	23 individuals	[43]
Association between pathology and clinical diagnosis	Each unit increase in AD pathology increases the likelihood of clinical AD diagnosis by nearly 20 times	Each unit increase in AD pathology triples the likelihood of clinical AD diagnosis	[43]

(Instances marked as ‘N/A’ do not indicate missing data but reflect either non-significant findings or a lesser degree of change in males relative to females).

#### 2.3.2. Sociological Factors

Sociological factors play a significant role in shaping gender differences in AD, influencing both cognitive outcomes and overall quality of life [76].

Educational attainment is a significant contributor to the risk of developing Alzheimer’s disease. Studies suggest that women with lower educational attainment are at greater risk for AD than men [77]. Limited education may reduce cognitive reserve, thereby increasing vulnerability to AD [33]. Gender inequality in occupational roles further compounds this risk. Women are more likely to be engaged in low-wage, low-autonomy occupations with limited cognitive stimulation, which may heighten the long-term risk of AD [78].

Depression is an independent risk factor for AD and also exhibits gender disparity. Epidemiological data indicate that women are nearly twice as likely as men to suffer from depression, with symptoms that are more persistent and prone to recurrence. On the one hand, prolonged depressive episodes can aggravate neuroinflammation and neuronal injury, thereby increasing the likelihood of AD onset. On the other hand, the decline in emotional regulation associated with AD makes patients more susceptible to depressive symptoms, thereby reinforcing a vicious cycle between mood disorder and disease progression [79].

In their roles as primary caregivers, women are more likely to experience chronic stress and disrupted sleep patterns due to the cumulative pressures of domestic responsibilities, professional obligations, and caregiving duties. Such psychosocial stressors have been shown to affect brain function negatively [58]. Notably, sleep disturbances—more prevalent in women—can hinder β-amyloid clearance, thereby promoting the accumulation of neuropathological changes associated with AD [80].

Unhealthy lifestyle factors, such as obesity, smoking, poor dietary habits, excessive alcohol consumption, and malnutrition, can all impair neural health in both AD women and men, accelerating brain atrophy and promoting tau protein deposition in specific brain regions, which disrupts the synthesis of neurotransmitter precursors [81,82]. While these factors affect both sexes, studies suggest that men are generally more likely to engage in behaviors such as smoking and heavy alcohol use, which are independently associated with elevated AD risk [83,84]. Notably, women appear more biologically vulnerable to the neurodegenerative effects of these exposures. For instance, chronic alcohol consumption exacerbates tau pathology, impairs lysosomal degradation, and promotes neuroinflammation to a greater extent in females than in males [85]. High and low body mass index (BMI) in middle and late life are associated with higher AD risk. A BMI over 29 kg/m^2^ in midlife increases AD risk, while maintaining a BMI below 27 kg/m^2^ in late life reduces it [86]. Interestingly, although abnormal BMI trajectories are linked to AD in both genders, studies show that weight loss and BMI variability in later life are more predictive of AD development in women [87]. A longitudinal study revealed that a 1.0-unit increase in BMI at age 70 correlates with a 36% higher AD risk in elderly women—a pattern not observed in men [88]. Critically, diet acts as a pivotal mediator in this BMI-AD relationship [89]. High-calorie diets rich in saturated fats promote Aβ deposition and accelerate brain atrophy, mechanistically linking obesity to AD neuropathology [90,91]. Conversely, diets abundant in polyphenols—found in berries—exert neuroprotective effects [92]. These compounds attenuate Aβ neuropathology by enhancing non-amyloidogenic amyloid β-protein processing, while their antioxidant and anti-inflammatory properties combat oxidative stress and neuroinflammation [93,94]. Notably, individuals adhering to a higher number of healthy lifestyle behaviors exhibit a markedly reduced risk of AD. Compared to individuals with 0–1 healthy lifestyle factors, those with 2–3 factors experience a 37% risk reduction, and those with 4–5 factors up to 60% [95]. Women aged 65 with four or five healthy behaviors have a life expectancy of 24.2 years, living 3.1 years longer than those with none or one factor (21.1 years). Moreover, they spend only 10.8% (2.6 years) of this period with AD, compared to 19.3% (4.1 years) in the low-lifestyle group. Similarly, men with four or five healthy factors have a life expectancy of 23.1 years—5.7 years longer than their counterparts—and spend 6.1% (1.4 years) of their remaining life with AD, versus 12.0% (2.1 years) in those with fewer healthy habits [96]. These findings underscore the critical importance of maintaining a healthy weight and adopting balanced dietary and lifestyle patterns in reducing gender-specific AD burden [97].

**Figure 1:**
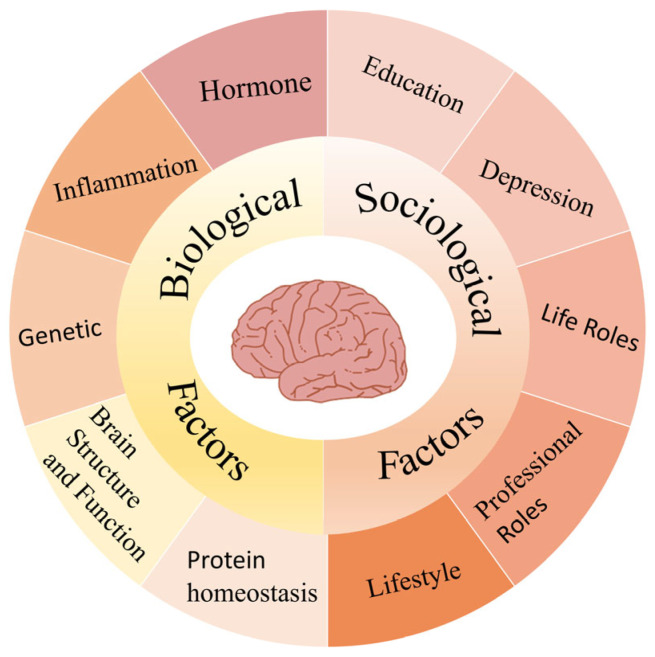
Gender-related factors involved in the development of Alzheimer’s disease.

In summary, gender disparities in Alzheimer’s disease arise from the combined effects of biological and sociological factors. Hormonal fluctuations, immune responses, and brain structural differences interact with educational level, caregiving roles, and mental health conditions, increasing disease vulnerability in women. These interconnected factors contribute to sex-specific patterns in AD development, highlighting the need for gender prevention and intervention strategies (Figure 1).

## 3. Gender-Specific Effects of Exercise Interventions on AD Risk

### 3.1. Baseline Gender Differences in Exercise Interventions

Given the pronounced gender disparities in the epidemiology, pathology, and clinical progression of AD, it is important to consider how these differences may influence or reflect broader patterns of physiological adaptation, including responses to physical exercise. Increasing evidence indicates that gender plays a crucial role in shaping the effects of exercise interventions on physical function in older adults [98].

Studies have shown that older women over 60 years old demonstrate greater cognitive improvements following various exercise programs, including aerobic training, resistance training, and multimodal training, with a mean cognitive gain of 1.32 points, compared to 0.98 points in men [99,100]. In particular, women experience more pronounced benefits in executive function, as evidenced by greater improvements on tests such as the Digit Symbol Substitution Test, Stroop interference, Trail Making Test Part B, and Task Switching [101,102]. Inflammatory responses also reflect gender-based differences in exercise interventions in older populations. After whole-body resistance training, changes in creatine kinase (CK) and IL-6 levels were significantly correlated in women but not in men, suggesting higher immunometabolic sensitivity in females [103]. Further clinical evidence reinforces this sex-specific responsiveness. A 12-week resistance training program in post–cardiac surgery patients confirmed the safety and feasibility of such interventions in older adults. Importantly, female participants showed greater relative improvements in grip strength, endurance, and functional capacity compared to males, despite similar baseline characteristics and adherence rates. These results suggest that older women may possess greater physiological adaptability to resistance training, underscoring the need for sex-informed approaches in exercise prescription [104].

Emerging evidence from structural and behavioral studies further supports these gender-based differences. Low-intensity daily walking activity, especially walking frequency and total exercise volume, is positively associated with hippocampal volume in women, but not in men. This suggests that women may derive more structural brain benefits from physical activity [105]. Gender-based differences in activity patterns may partially explain these findings: women are more likely to engage in domestic and light-intensity activities, whereas men tend to participate in sports or structured exercise, potentially influencing the magnitude and type of benefits observed [106]. In interventions involving exercise intensity and duration, postmenopausal women have demonstrated greater improvements in cardiovascular markers such as Low-Density Lipoprotein (LDL) and cholesterol after high-intensity interval training (HIIT) compared to men. These differences highlight the importance of tailoring exercise prescriptions to gender-specific physiological profiles [107]. Longitudinal studies show that maintaining physical activity over 10 years is associated with slower executive function decline in women, whereas men do not show the same sustained benefit [108]. A review on gender differences in the aging brain reported that progressive, moderate-intensity aerobic training resulted in a 36% improvement in executive function in women, whereas men showed a 31% decline. Notably, this effect persisted for 6 months, underscoring the potential importance of incorporating gender considerations into exercise interventions. However, the review also highlighted that not all studies demonstrated a female advantage. For example, in a 12-month study of moderate-intensity aerobic training, sex-stratified analyses indicated that improved exercise compliance was associated with improvements in attention and memory in older women with MCI, but also with memory gains in older men. These findings suggest that the gender-specific effects of exercise on cognition may be influenced by individual factors such as state and compliance, highlighting the necessity of further research on the consistency and mechanisms of gender differences [109,110]. In terms of overall function, although older men showed improvements in general strength and cardiorespiratory fitness following aerobic, strength, or combined training, when considering all health indicators, exercise was found to have a more pronounced positive impact on women’s overall health [111].

Animal studies further support the gender differences observed in human experiments. Barha et al. found that healthy elderly female mice improved learning and memory following aerobic training, particularly in hippocampus-dependent tasks, where female mice outperformed male mice in cognitive performance. In contrast, males exhibited more substantial autonomous training effects in non-spatial memory tasks, while females performed better under forced training, especially in hippocampus-related tasks [112]. Furthermore, resistance training improved intramuscular mitochondrial density, oxidative capacity, and autophagic activity in male mice, while female mice showed more significant increases in heart mass [113]. These findings suggest that exercise interventions may enhance neural adaptations in different genders through distinct mechanisms.

A growing body of evidence suggests that older women may tend to experience more pronounced benefits from physical exercise than men, particularly in domains such as cognitive function, emotional well-being, and neural structure. These gender-related advantages have been observed across various exercise modalities and intensities, especially in aerobic and moderate-to-high-intensity interventions. However, it is important to note that these findings are based on the specific studies included in this review and may not reflect a universal consensus across the broader literature. The limited number of studies directly comparing gender-specific responses to different exercise protocols underscores the need for more comprehensive, large-scale investigations to validate these patterns and determine their generalizability.

### 3.2. Gender-Specific Manifestations of Exercise Intervention in AD Pathological Scenarios

The observed baseline differences in exercise responsiveness between men and women serve as a crucial basis for understanding how gender may influence the outcomes of interventions in the context of Alzheimer’s disease. Given the biological mechanisms shared by normal aging and AD—such as neuroinflammation, impaired neurotrophic signaling, and metabolic dysregulation—it is plausible that gender-specific patterns seen in cognitively healthy older adults persist or even intensify as the disease advances. Notably, individuals with AD display more intricate gender-related variations in response to exercise, particularly in domains such as neural function, pathological progression, and emotional regulation [114].

At the stage of cognitive impairment, women appear to experience greater cognitive benefits from exercise compared to men. Barha et al. found that, following aerobic training, female patients showed significant improvements in cognitive tasks such as the Trail Making Test (TMT), digit span, and Stroop test. In contrast, male patients did not exhibit comparable gains, suggesting that aerobic exercise may have more pronounced effects on cognitive performance in women [115]. A six-month aerobic exercise intervention further validated these observations. Following the intervention, female AD patients showed significant improvements across multiple domains in seven cognitive assessments, whereas male participants demonstrated gains in only one [116].

In the emotional and behavioral responses domain, AD patients show distinct gender-based differences in their reactions to physical activity. Stojanovic et al. found that exercise (measured by the Physical Activity Scale for the Elderly) in female patients not only led to marked improvements in memory and attention but also effectively alleviated symptoms of anxiety and depression, thereby facilitating broader cognitive enhancement. In contrast, while male patients exhibited some memory improvement, the association between emotional benefits and exercise participation was less pronounced, indicating that women with AD may be more responsive to the emotion-regulating effects of physical activity [117]. Findings from animal studies further substantiate these conclusions. In 3xTg-AD mice subjected to 6 months of voluntary wheel running, male mice exhibited higher overall activity levels during exercise. However, female mice demonstrated greater benefits in terms of weight regulation and cognitive performance. Specifically, in the light/dark box test, female mice showed reduced anxiety-like behavior, as evidenced by a shorter latency to enter the light area. In the Morris water maze, exercise improved spatial learning and memory, as shown by decreased escape latency during acquisition of the place task and increased time spent in the platform quadrant during the probe trial. These effects were more pronounced in female mice, suggesting a greater delay in cognitive decline. Together, the results indicate that while male mice are more physically active, female mice may experience greater neurocognitive and emotional benefits from long-term voluntary exercise [118]. Moreover, treadmill exercise reduced depressive behaviors in female AD-model rats, whereas improvements in male counterparts were comparatively limited [119].

### 3.3. Mechanisms Underlying Gender Differences in Exercise Effects on AD: Evidence from Human and Animal Studies

To better understand the gender-specific effects of exercise in individuals with Alzheimer’s disease, a more in-depth investigation is needed into the physiological processes and hormonal regulatory pathways that may underlie these differences [120].

Clinical studies have demonstrated gender-specific endocrine and neurotrophic responses to aerobic exercise in individuals with AD. Among female patients, aerobic training leads to an increase in BDNF levels, which is closely related to enhanced neural plasticity. In contrast, the BDNF level of male patients showed a downward trend [115]. Moreover, aerobic exercise in females results in a significant reduction in fasting plasma insulin and cortisol levels, along with improved glucose utilization, suggesting enhanced metabolic and stress-regulatory adaptations. Conversely, male participants primarily exhibit increased levels of insulin-like growth factor-1 (IGF-1), indicating distinct hormonal response pathways that may underlie gender differences in the cognitive and neuroprotective effects of exercise [116].

Evidence from animal models similarly suggests that running exercise induces more substantial neuroprotective effects in females, particularly in terms of structural and functional optimization of white matter. In early-stage AD mice, females exhibit significant increases in myelinated fiber diameter and total length following physical training, in contrast to males, indicating a more favorable response to exercise during the initial stages of pathology [121].

In terms of neuroinflammation regulation, exercise led to reductions in both CD86^+^ and MHCII^+^ microglial populations in the hippocampus of aged female mice, while in males, only CD86^+^ microglia decreased, and MHCII^+^ levels increased. This pattern suggests that running wheel exercise more effectively suppresses neuroinflammatory responses and enhances antigen tolerance in females, thereby promoting neural homeostasis and brain health [122]. In addition, in a study using htau transgenic mice, administration of recombinant irisin beginning at a pre-symptomatic stage significantly reduced hippocampal phosphorylated tau and tumor necrosis factor-α levels in females, while no such reduction was observed in males. In fact, irisin treatment in males appeared to increase both central and peripheral TNF-α concentrations, suggesting a potential sex-dependent inflammatory response to irisin [123]. These findings underscore the greater potential of exercise to mitigate neuroinflammatory burden in females.

Building on the inflammatory differences, gender-specific effects extend further into neurotrophic and hormonal signaling pathways. Voluntary wheel running induced a more pronounced increase in BDNF levels in female mice, supporting neuronal maintenance and synaptic plasticity [124]. Herring et al. further demonstrated that running exercise enhanced angiogenesis and attenuated microglial proliferation in female mice, reducing cerebral inflammation and Aβ plaque burden. These effects could be linked to the neuroprotective actions of estrogen released during physical activity [125]. This notion is supported by findings from ovariectomy models, in which voluntary exercise in a running wheel restored cognitive and behavioral function through BDNF upregulation and cyclic AMP response element-binding protein (CREB) pathway activation [126]. In contrast, although Aβ levels showed minimal changes in male mice following aerobic fitness intervention, androgens were critical in mediating improvements in tau pathology and plasma oxidative capacity [127].

Additionally, a study by Giménez-Llort et al. on the 3xTg-AD mouse model revealed gender-specific adaptations to 5 weeks of treadmill training. In male mice, exercise reduced oxidative stress and ameliorated GABA-A receptor dysfunction, while female mice display more marked improvements in sensorimotor function. Exercise decreased the Aβ42/40 ratio in both sexes, suggesting a shared beneficial effect on AD-related pathology. However, mechanistic analyses indicated divergent regulatory pathways between males and females, highlighting gender-dependent modulation of disease progression [128].

In genetic AD mouse models, treadmill exercise significantly improved spatial learning and memory performance in male mice while effectively reducing levels of the inflammatory marker IL-6 in the hippocampus of female mice [129]. These findings suggest that males and females may derive distinct functional benefits from exercise due to differences in gene expression profiles, reflecting gender-specific molecular response mechanisms.

Although exercise exerts multifaceted benefits in Alzheimer’s disease, pronounced gender differences are evident in both its effects and underlying mechanisms. Females generally experience greater improvements in cognition and emotional regulation, whereas males display distinct responses in metabolic and hormonal pathways (Table 3).

## 4. Conclusions

In summary, gender plays a fundamental role throughout the onset and progression of AD. Compared to men, women are generally at higher risk and often present with more severe clinical manifestations. These disparities arise from a complex interplay of biological, psychosocial, and lifestyle-related factors, including hormonal shifts, immune reactivity, structural and functional brain differences, genetic predispositions, and social roles. Physical exercise, as a non-pharmacological intervention, has been shown to delay cognitive decline by enhancing BDNF expression, reducing neuroinflammation, and regulating hormonal balance. Additionally, exercise improves white matter integrity and psychological resilience, especially among female patients.

This review provides a general overview of gender-specific responses to exercise in the context of Alzheimer’s disease. However, several limitations should be acknowledged. First, the exercise protocols varied considerably across studies, including differences in intensity, duration, and modality. In some cases, the intervention parameters were not clearly reported, making direct comparison across studies difficult. Second, the number of studies that directly examine the gender-specific effects of exercise on AD remains limited, especially in clinical populations, which restricts the amount of evidence available for analysis. Third, many of the findings discussed are based on animal models under controlled laboratory settings, which may not fully reflect the complexity of gender-related mechanisms in humans with AD. Finally, although this review highlights the potential benefits of exercise by gender, the lack of dose–response data across AD stages prevents us from proposing optimized exercise strategies tailored to gender or disease progression.

Future research should focus on elucidating the molecular mechanisms through which gender differences influence the outcomes of exercise interventions in AD. Randomized controlled trials are essential to develop and validate personalized exercise protocols tailored to male and female patients, ensuring a more precise evaluation of intervention efficacy. Moreover, investigating the combined effects of exercise type and intensity across different age groups may provide further insight into gender-specific responses. Studies exploring the integration of exercise with pharmacological and nutritional approaches could also offer promising strategies for gender-based AD prevention and management.

## Figures and Tables

**Table 3:** Experimental models, exercise protocols, and gender-specific effects in AD.

Experimental Model	Type of Physical Exercise	Protocol	Exercise Intensity	Duration	Number of Days Per Week	Effects	Reference
Human (≥55 years)	Aerobic training	Classes were 60 min in duration (10 min warm-up, 40 min walking, 10 min cool down)	Moderate, progressed to 60–70% HRR	6 months	3 times per week	Female: ↑ executive function (set-shifting), ↑ BDNF; male: ↑ functional fitness	[115]
33 Participants	Treadmill, stationary bicycle, elliptical trainer	45 to 60 min per session	Progressive to 75–85% HRR	6 months	4 times per week	Female: ↑ executive function, ↑ glucose disposal, ↓ insulin, cortisol, BDNF; male:↑ IGF-1, slight cognitive improvement (Trails B)	[116]
Participants (aged 61 to 92 years old)	N/A	Physical Activity Scale for the Elderly	N/A	N/A	N/A	Female: mood was a significant predictor of physical activity engagement; male: physical activity levels are correlated with ptau181 accumulation	[117]
3xTg-AD mice	Wheel running	Experiment I: 1 month (starting at 3 months of age, ending at 4 months) Experiment II: 1 month or 6 months (starting at 6 or 1 month of age, ending at 7 months) Experiment III: 6 months (starting at 1 month of age, ending at 7 months)	voluntary	6 months	N/A	Female: Greater or comparable benefit in AD-related pathology and behavior; male: higher oxidative stress. Both sexes: ↓ cognitive decline, ↓ anxiety/startle response, ↑ antioxidant defense, partial synaptic protection	[118]
80 adult male and female Wistar rats	Treadmill exercise	a running session (30 min) per day, “warm-up” period of 7 min at 5 m/min, “running” 16 min at 10 m/min (weeks 0–7) or 12 m/min (weeks 7–12), and a “cool-up” period of 7 min at 5 m/min	N/A	12 weeks	5 days per week	Female: ↑ BDNF (hippocampus), ↑ IL-10 (prefrontal cortex), ↓ TNF-α (hippocampus and PFC), ↓ depressive-like behaviors (including anhedonia), ↓ body weight and food intake; male: ↓ TNF-α (hippocampus), ↓ anhedonia-like behavior, ↓ body weight and food intake; no significant ↑ in BDNF or other cytokines; limited behavioral improvement	[119]
APP/PS1 double transgenic mice	Treadmill running	Adaptation period: One week (5 m/min, 10 min/day), training period: 10 m/min, 20 min/day	N/A	4 months	6 days per week	Female: Greater improvements in spatial learning and memory, ↑ white matter volume and myelinated fiber parameters; male: Less pronounced changes	[121]
BALB/c mice	Wheel running	N/A	N/A	Experiment 1: 10 weeks; Experiment 2: 8 weeks	N/A	Female: ↓ CD86^+^ and MHC II^+^ microglia in hippocampus; male: ↓ CD86^+^ in brain, ↑ MHC II^+^ in hippocampus and brain	[122]
C57Bl/6J mice	Wheel running	3 months access from age 8–11 months	Voluntary	3 months	N/A	Female: Conditional fear has no effect, ↑ BDNF mRNA expression; male: Recovery of conditional fear ability, ↓ decline in cognitive ability, ↑ expression of BDNF mRNA	[124]
Offspring of APP transgenic mice	Running exercise	Mice ran an average of 0.63 ± 0.08 km daily	Voluntary	N/A	daily	Female: Exercise reduced Aβ burden and inflammation in offspring; improved vascular function and synaptic plasticity	[125]
3xTg—AD mice	Running wheel exercise	N/A	Voluntary	3 months	N/A	Female: Exercise protected against memory loss, apathy, BPSD-like behaviors, and frailty; partially restored BDNF-CREB signaling	[126]
120 Participants	Aerobic fitness	≥10 years of aerobic training, 150 min/week	Moderate (Borg Rating and Perceived Exertion scale)	N/A	N/A	Female: ↓ erythrocyte β-amyloid, ↑ plasma antioxidant capacity; male: ↓ tau levels in erythrocytes; ↑ plasma antioxidant capacity	[127]
3xTg-AD mice	Treadmill training	Gradual treadmill training: 15 → 30 min/day, 5 → 7 cm/s	Low to moderate	5 weeks	5 days per week	Female: ↑ sensorimotor function; male: ↓ oxidative stress, ↓ GABA-A receptor dysfunction; Both sexes: ↓ Aβ42/40 ratio	[128]
GFAP-ApoE3 and GFAP-ApoE4 mice	Treadmill training	Progressive treadmill training to 1 h/day over 12 days (up to 14 m/min, 40 min)	N/A	8 weeks	N/A	Female: Exercise improved activity, learning, and memory in GFAP-ApoE3 females; reduced IL-6 in GFAP-ApoE3 females, but increased TNFα in GFAP-ApoE4 females; showed better bridge walking and discrimination task performance under Ex-Aox condition; male: Exercise improved motor coordination, learning (Morris Water Maze), and GABA-related function in GFAP-ApoE3 males; less pronounced cognitive gains in GFAP-ApoE4 males	[129]

(The ‘N/A’ in Table 3 indicates that the corresponding information is not mentioned in the cited literature. In the “Effects” column of Table 3, “↑” indicates an increase or improvement, while “↓” indicates a decrease or aggravation).

## Data Availability

Not applicable.

## Abbreviations

The following abbreviations are used in this manuscript:

AD	Alzheimer’s disease
Aβ	amyloid-beta
NFT	neurofibrillary tangles
MCI	mild cognitive impairment
NPS	neuropsychiatric symptoms
APOE4	Apolipoprotein E4
T-tau	total tau
P-tau	phosphorylated tau
SCD	subjective cognitive decline
SMC	subjective memory complaints
BDNF	brain-derived neurotrophic factor
HRT	hormone replacement therapy
1L-1β	Interleukin-1β
1L-6	Interleukin-6
BMI	body mass index
CK	creatine kinase
LDL	low-Denisty Lipoprotein
HIIT	high-intensity interval training
TMT	Trail Making Test
IGF-1	insulin-like growth factor-1
TNF-α	tumor necrosis factor-alpha
CREB	cyclic AMP response element-binding

## References

[1] Stefaniak O., Dobrzyńska M., Drzymała-Czyż S., Przysławski J. (2022). Diet in the Prevention of Alzheimer’s Disease: Current Knowledge and Future Research Requirements. Nutrients.

[2] Podcasy J.L., Epperson C.N. (2016). Considering sex and gender in Alzheimer disease and other dementias. Dialogues Clin. Neurosci..

[3] Kheloui S., Jacmin-Park S., Larocque O., Kerr P., Rossi M., Cartier L., Juster R.P. (2023). Sex/gender differences in cognitive abilities. Neurosci. Biobehav. Rev..

[4] Rosende-Roca M., García-Gutiérrez F., Cantero-Fortiz Y., Alegret M., Pytel V., Cañabate P., González-Pérez A., de Rojas I., Vargas L., Tartari J.P. (2025). Exploring sex differences in Alzheimer’s disease: A comprehensive analysis of a large patient cohort from a memory unit. Alzheimers Res. Ther..

[5] Mishra P., Davies D.A., Albensi B.C. (2023). The Interaction Between NF-κB and Estrogen in Alzheimer’s Disease. Mol. Neurobiol..

[6] Yang Z., Levey A. (2015). Gender Differences: A Lifetime Analysis of the Economic Burden of Alzheimer’s Disease. Womens Health Issues.

[7] (2021). 2021 Alzheimer’s disease facts and figures. Alzheimers Dement..

[8] Birks J.S., Harvey R.J. (2018). Donepezil for dementia due to Alzheimer’s disease. Cochrane Database Syst. Rev..

[9] Reisberg B., Doody R., Stöffler A., Schmitt F., Ferris S., Möbius H.J. (2003). Memantine in moderate-to-severe Alzheimer’s disease. N. Engl. J. Med..

[10] van Dyck C.H., Swanson C.J., Aisen P., Bateman R.J., Chen C., Gee M., Kanekiyo M., Li D., Reyderman L., Cohen S. (2023). Lecanemab in Early Alzheimer’s Disease. N. Engl. J. Med..

[11] Dou K.X., Tan M.S., Tan C.C., Cao X.P., Hou X.H., Guo Q.H., Tan L., Mok V., Yu J.T. (2018). Comparative safety and effectiveness of cholinesterase inhibitors and memantine for Alzheimer’s disease: A network meta-analysis of 41 randomized controlled trials. Alzheimers Res. Ther..

[12] Whittington M.D., Campbell J.D., Rind D., Fluetsch N., Lin G.A., Pearson S.D. (2022). Cost-Effectiveness and Value-Based Pricing of Aducanumab for Patients With Early Alzheimer Disease. Neurology.

[13] López-Ortiz S., Lista S., Valenzuela P.L., Pinto-Fraga J., Carmona R., Caraci F., Caruso G., Toschi N., Emanuele E., Gabelle A. (2023). Effects of physical activity and exercise interventions on Alzheimer’s disease: An umbrella review of existing meta-analyses. J. Neurol..

[14] Valenzuela P.L., Castillo-García A., Morales J.S., de la Villa P., Hampel H., Emanuele E., Lista S., Lucia A. (2020). Exercise benefits on Alzheimer’s disease: State-of-the-science. Ageing Res. Rev..

[15] Cámara-Calmaestra R., Martínez-Amat A., Aibar-Almazán A., Hita-Contreras F., de Miguel Hernando N., Achalandabaso-Ochoa A. (2022). Effectiveness of Physical Exercise on Alzheimer’s disease. A Systematic Review. J. Prev. Alzheimers Dis..

[16] Sepúlveda-Lara A., Sepúlveda P., Marzuca-Nassr G.N. (2024). Resistance Exercise Training as a New Trend in Alzheimer’s Disease Research: From Molecular Mechanisms to Prevention. Int. J. Mol. Sci..

[17] Coutinho L.A., Leão L.L., Cassilhas R.C., de Paula A.M.B., Deslandes A.C., Monteiro-Junior R.S. (2022). Alzheimer’s disease genes and proteins associated with resistance and aerobic training: An in silico analysis. Exp. Gerontol..

[18] Tsai C.L., Pai M.C., Ukropec J., Ukropcová B. (2019). Distinctive Effects of Aerobic and Resistance Exercise Modes on Neurocognitive and Biochemical Changes in Individuals with Mild Cognitive Impairment. Curr. Alzheimer Res..

[19] Stefanacci R.G. (2011). The costs of Alzheimer’s disease and the value of effective therapies. Am. J. Manag. Care.

[20] (2024). 2024 Alzheimer’s disease facts and figures. Alzheimers Dement..

[21] Gustavsson A., Norton N., Fast T., Frölich L., Georges J., Holzapfel D., Kirabali T., Krolak-Salmon P., Rossini P.M., Ferretti M.T. (2023). Global estimates on the number of persons across the Alzheimer’s disease continuum. Alzheimers Dement..

[22] Rajan K.B., Weuve J., Barnes L.L., McAninch E.A., Wilson R.S., Evans D.A. (2021). Population estimate of people with clinical Alzheimer’s disease and mild cognitive impairment in the United States (2020–2060). Alzheimers Dement..

[23] Ji Q., Chen J., Li Y., Tao E., Zhan Y. (2024). Incidence and prevalence of Alzheimer’s disease in China: A systematic review and meta-analysis. Eur. J. Epidemiol..

[24] Seshadri S., Wolf P.A., Beiser A., Au R., McNulty K., White R., D’Agostino R.B. (1997). Lifetime risk of dementia and Alzheimer’s disease. The impact of mortality on risk estimates in the Framingham Study. Neurology.

[25] Hebert L.E., Scherr P.A., McCann J.J., Beckett L.A., Evans D.A. (2001). Is the risk of developing Alzheimer’s disease greater for women than for men?. Am. J. Epidemiol..

[26] Wang X., Feng S., Deng Q., Wu C., Duan R., Yang L. (2025). The role of estrogen in Alzheimer’s disease pathogenesis and therapeutic potential in women. Mol. Cell Biochem..

[27] Ma C., Hong F., Yang S. (2022). Amyloidosis in Alzheimer’s Disease: Pathogeny, Etiology, and Related Therapeutic Directions. Molecules.

[28] Arenaza-Urquijo E.M., Boyle R., Casaletto K., Anstey K.J., Vila-Castelar C., Colverson A., Palpatzis E., Eissman J.M., Kheng Siang Ng T., Raghavan S. (2024). Sex and gender differences in cognitive resilience to aging and Alzheimer’s disease. Alzheimers Dement..

[29] Nebel R.A., Aggarwal N.T., Barnes L.L., Gallagher A., Goldstein J.M., Kantarci K., Mallampalli M.P., Mormino E.C., Scott L., Yu W.H. (2018). Understanding the impact of sex and gender in Alzheimer’s disease: A call to action. Alzheimers Dement..

[30] Liesinger A.M., Graff-Radford N.R., Duara R., Carter R.E., Hanna Al-Shaikh F.S., Koga S., Hinkle K.M., DiLello S.K., Johnson M.F., Aziz A. (2018). Sex and age interact to determine clinicopathologic differences in Alzheimer’s disease. Acta Neuropathol..

[31] Corder E.H., Ghebremedhin E., Taylor M.G., Thal D.R., Ohm T.G., Braak H. (2004). The biphasic relationship between regional brain senile plaque and neurofibrillary tangle distributions: Modification by age, sex, and APOE polymorphism. Ann. N. Y. Acad. Sci..

[32] Coughlan G.T., Klinger H.M., Boyle R., Betthauser T.J., Binette A.P., Christenson L., Chadwick T., Hansson O., Harrison T.M., Healy B. (2025). Sex Differences in Longitudinal Tau-PET in Preclinical Alzheimer Disease: A Meta-Analysis. JAMA Neurol..

[33] Mielke M.M., Aggarwal N.T., Vila-Castelar C., Agarwal P., Arenaza-Urquijo E.M., Brett B., Brugulat-Serrat A., DuBose L.E., Eikelboom W.S., Flatt J. (2022). Consideration of sex and gender in Alzheimer’s disease and related disorders from a global perspective. Alzheimers Dement..

[34] Callahan M.J., Lipinski W.J., Bian F., Durham R.A., Pack A., Walker L.C. (2001). Augmented senile plaque load in aged female beta-amyloid precursor protein-transgenic mice. Am. J. Pathol..

[35] Emrani S., Sundermann E.E. (2025). Sex/gender differences in the clinical trajectory of Alzheimer’s disease: Insights into diagnosis and cognitive reserve. Front. Neuroendocrinol..

[36] Sohn D., Shpanskaya K., Lucas J.E., Petrella J.R., Saykin A.J., Tanzi R.E., Samatova N.F., Doraiswamy P.M. (2018). Sex Differences in Cognitive Decline in Subjects with High Likelihood of Mild Cognitive Impairment due to Alzheimer’s disease. Sci. Rep..

[37] Irvine K., Laws K.R., Gale T.M., Kondel T.K. (2012). Greater cognitive deterioration in women than men with Alzheimer’s disease: A meta analysis. J. Clin. Exp. Neuropsychol..

[38] Ardekani B.A., Hadid S.A., Blessing E., Bachman A.H. (2019). Sexual Dimorphism and Hemispheric Asymmetry of Hippocampal Volumetric Integrity in Normal Aging and Alzheimer Disease. AJNR Am. J. Neuroradiol..

[39] Koran M.E.I., Wagener M., Hohman T.J. (2017). Sex differences in the association between AD biomarkers and cognitive decline. Brain Imaging Behav..

[40] Sundermann E.E., Maki P.M., Rubin L.H., Lipton R.B., Landau S., Biegon A. (2016). Female advantage in verbal memory: Evidence of sex-specific cognitive reserve. Neurology.

[41] Pérès K., Helmer C., Amieva H., Matharan F., Carcaillon L., Jacqmin-Gadda H., Auriacombe S., Orgogozo J.M., Barberger-Gateau P., Dartigues J.F. (2011). Gender differences in the prodromal signs of dementia: Memory complaint and IADL-restriction. a prospective population-based cohort. J. Alzheimers Dis..

[42] Eikelboom W.S., Pan M., Ossenkoppele R., Coesmans M., Gatchel J.R., Ismail Z., Lanctôt K.L., Fischer C.E., Mortby M.E., van den Berg E. (2022). Sex differences in neuropsychiatric symptoms in Alzheimer’s disease dementia: A meta-analysis. Alzheimers Res. Ther..

[43] Barnes L.L., Wilson R.S., Bienias J.L., Schneider J.A., Evans D.A., Bennett D.A. (2005). Sex differences in the clinical manifestations of Alzheimer disease pathology. Arch. Gen. Psychiatry.

[44] Wang X. Analysis on Risk Factors of Women in Alzheimer’s Disease. Proceedings of the 2020 International Conference on Public Health and Data Science (ICPHDS).

[45] Li R., Singh M. (2014). Sex differences in cognitive impairment and Alzheimer’s disease. Front. Neuroendocrinol..

[46] Lee B.H., Eid R.S., Hodges T.E., Barth C., Galea L.A.M. (2025). Leveraging research into sex differences and steroid hormones to improve brain health. Nat. Rev. Endocrinol..

[47] Bagit A., Hayward G.C., MacPherson R.E.K. (2021). Exercise and estrogen: Common pathways in Alzheimer’s disease pathology. Am. J. Physiol. Endocrinol. Metab..

[48] Rettberg J.R., Yao J., Brinton R.D. (2014). Estrogen: A master regulator of bioenergetic systems in the brain and body. Front. Neuroendocrinol..

[49] Scheyer O., Rahman A., Hristov H., Berkowitz C., Isaacson R.S., Diaz Brinton R., Mosconi L. (2018). Female Sex and Alzheimer’s Risk: The Menopause Connection. J. Prev. Alzheimers Dis..

[50] Rahman A., Schelbaum E., Hoffman K., Diaz I., Hristov H., Andrews R., Jett S., Jackson H., Lee A., Sarva H. (2020). Sex-driven modifiers of Alzheimer risk: A multimodality brain imaging study. Neurology.

[51] Lv W., Du N., Liu Y., Fan X., Wang Y., Jia X., Hou X., Wang B. (2016). Low Testosterone Level and Risk of Alzheimer’s Disease in the Elderly Men: A Systematic Review and Meta-Analysis. Mol. Neurobiol..

[52] Gouras G.K., Xu H., Gross R.S., Greenfield J.P., Hai B., Wang R., Greengard P. (2000). Testosterone reduces neuronal secretion of Alzheimer’s beta-amyloid peptides. Proc. Natl. Acad. Sci. USA.

[53] Hogervorst E., Bandelow S., Combrinck M., Smith A.D. (2004). Low free testosterone is an independent risk factor for Alzheimer’s disease. Exp. Gerontol..

[54] Cherrier M.M., Matsumoto A.M., Amory J.K., Asthana S., Bremner W., Peskind E.R., Raskind M.A., Craft S. (2005). Testosterone improves spatial memory in men with Alzheimer disease and mild cognitive impairment. Neurology.

[55] Tan R.S., Pu S.J. (2003). A pilot study on the effects of testosterone in hypogonadal aging male patients with Alzheimer’s disease. Aging Male.

[56] Marriott R.J., Murray K., Flicker L., Hankey G.J., Matsumoto A.M., Dwivedi G., Antonio L., Almeida O.P., Bhasin S., Dobs A.S. (2022). Lower serum testosterone concentrations are associated with a higher incidence of dementia in men: The UK Biobank prospective cohort study. Alzheimers Dement..

[57] Sundermann E.E., Panizzon M.S., Chen X., Andrews M., Galasko D., Banks S.J. (2020). Sex differences in Alzheimer’s-related Tau biomarkers and a mediating effect of testosterone. Biol. Sex Differ..

[58] Zhu D., Montagne A., Zhao Z. (2021). Alzheimer’s pathogenic mechanisms and underlying sex difference. Cell Mol. Life Sci..

[59] Casaletto K.B., Nichols E., Aslanyan V., Simone S.M., Rabin J.S., La Joie R., Brickman A.M., Dams-O’Connor K., Palta P., Kumar R.G. (2022). Sex-specific effects of microglial activation on Alzheimer’s disease proteinopathy in older adults. Brain.

[60] Block M.L., Zecca L., Hong J.S. (2007). Microglia-mediated neurotoxicity: Uncovering the molecular mechanisms. Nat. Rev. Neurosci..

[61] Heneka M.T., Kummer M.P., Latz E. (2014). Innate immune activation in neurodegenerative disease. Nat. Rev. Immunol..

[62] Biechele G., Rauchmann B.S., Janowitz D., Buerger K., Franzmeier N., Weidinger E., Guersel S., Schuster S., Finze A., Harris S. (2024). Associations between sex, body mass index and the individual microglial response in Alzheimer’s disease. J. Neuroinflammation.

[63] Guillot-Sestier M.V., Araiz A.R., Mela V., Gaban A.S., O’Neill E., Joshi L., Chouchani E.T., Mills E.L., Lynch M.A. (2021). Microglial metabolism is a pivotal factor in sexual dimorphism in Alzheimer’s disease. Commun. Biol..

[64] O’Neill E., Mela V., Gaban A.S., Bechet S., McGrath A., Walsh A., McIntosh A., Lynch M.A. (2022). Sex-Related Microglial Perturbation Is Related to Mitochondrial Changes in a Model of Alzheimer’s Disease. Front. Cell Neurosci..

[65] Chen Y., Hong T., Chen F., Sun Y., Wang Y., Cui L. (2021). Interplay Between Microglia and Alzheimer’s Disease-Focus on the Most Relevant Risks: APOE Genotype, Sex and Age. Front. Aging Neurosci..

[66] Sanfilippo C., Castrogiovanni P., Imbesi R., Vecchio M., Sortino M., Musumeci G., Vinciguerra M., Di Rosa M. (2025). Exploring SERPINA3 as a neuroinflammatory modulator in Alzheimer’s disease with sex and regional brain variations. Metab. Brain Dis..

[67] Zhang R., Xu X., Yu H., Xu X., Wang M., Le W. (2022). Factors Influencing Alzheimer’s Disease Risk: Whether and How They are Related to the APOE Genotype. Neurosci. Bull..

[68] Belloy M.E., Napolioni V., Greicius M.D. (2019). A Quarter Century of APOE and Alzheimer’s Disease: Progress to Date and the Path Forward. Neuron.

[69] Sampedro F., Vilaplana E., de Leon M.J., Alcolea D., Pegueroles J., Montal V., Carmona-Iragui M., Sala I., Sánchez-Saudinos M.B., Antón-Aguirre S. (2015). APOE-by-sex interactions on brain structure and metabolism in healthy elderly controls. Oncotarget.

[70] Edmunds K.J., Pandos A.A., Hoang I., Mamlouk G.M., Motovylyak A., Lose S.R., Asthana S., Stremlau M., Johnson S.C., van Praag H. (2025). BDNF expression mediates verbal learning and memory in women in a cohort enriched with risk for Alzheimer’s disease. Alzheimers Dement..

[71] Ritchie S.J., Cox S.R., Shen X., Lombardo M.V., Reus L.M., Alloza C., Harris M.A., Alderson H.L., Hunter S., Neilson E. (2018). Sex Differences in the Adult Human Brain: Evidence from 5216 UK Biobank Participants. Cereb. Cortex.

[72] Ingalhalikar M., Smith A., Parker D., Satterthwaite T.D., Elliott M.A., Ruparel K., Hakonarson H., Gur R.E., Gur R.C., Verma R. (2014). Sex differences in the structural connectome of the human brain. Proc. Natl. Acad. Sci. USA.

[73] Lopez-Lee C., Torres E.R.S., Carling G., Gan L. (2024). Mechanisms of sex differences in Alzheimer’s disease. Neuron.

[74] Dubal D.B. (2020). Sex difference in Alzheimer’s disease: An updated, balanced and emerging perspective on differing vulnerabilities. Handb. Clin. Neurol..

[75] Sultana O.F., Bandaru M., Islam M.A., Reddy P.H. (2024). Unraveling the complexity of human brain: Structure, function in healthy and disease states. Ageing Res. Rev..

[76] Rahman A., Jackson H., Hristov H., Isaacson R.S., Saif N., Shetty T., Etingin O., Henchcliffe C., Brinton R.D., Mosconi L. (2019). Sex and Gender Driven Modifiers of Alzheimer’s: The Role for Estrogenic Control Across Age, Race, Medical, and Lifestyle Risks. Front. Aging Neurosci..

[77] Subramaniapillai S., Almey A., Natasha Rajah M., Einstein G. (2021). Sex and gender differences in cognitive and brain reserve: Implications for Alzheimer’s disease in women. Front. Neuroendocrinol..

[78] Geraets A.F.J., Leist A.K. (2023). Sex/gender and socioeconomic differences in modifiable risk factors for dementia. Sci. Rep..

[79] Sramek J.J., Murphy M.F., Cutler N.R. (2016). Sex differences in the psychopharmacological treatment of depression. Dialogues Clin. Neurosci..

[80] Jee H.J., Shin W., Jung H.J., Kim B., Lee B.K., Jung Y.S. (2020). Impact of Sleep Disorder as a Risk Factor for Dementia in Men and Women. Biomol. Ther..

[81] Franke K., Ristow M., Gaser C. (2014). Gender-specific impact of personal health parameters on individual brain aging in cognitively unimpaired elderly subjects. Front. Aging Neurosci..

[82] Ye K.X., Sun L., Wang L., Khoo A.L.Y., Lim K.X., Lu G., Yu L., Li C., Maier A.B., Feng L. (2023). The role of lifestyle factors in cognitive health and dementia in oldest-old: A systematic review. Neurosci. Biobehav. Rev..

[83] Davuluri S., Bajpai A.K., Thirumurugan K., Acharya K.K. (2021). The molecular basis of gender disparities in smoking lung cancer patients. Life Sci..

[84] Schwarzinger M., Pollock B.G., Hasan O.S.M., Dufouil C., Rehm J. (2018). Contribution of alcohol use disorders to the burden of dementia in France 2008-13: A nationwide retrospective cohort study. Lancet Public Health.

[85] Tucker A.E., Alicea Pauneto C.D.M., Barnett A.M., Coleman L.G. (2022). Chronic Ethanol Causes Persistent Increases in Alzheimer’s Tau Pathology in Female 3xTg-AD Mice: A Potential Role for Lysosomal Impairment. Front. Behav. Neurosci..

[86] Qu Y., Hu H.Y., Ou Y.N., Shen X.N., Xu W., Wang Z.T., Dong Q., Tan L., Yu J.T. (2020). Association of body mass index with risk of cognitive impairment and dementia: A systematic review and meta-analysis of prospective studies. Neurosci. Biobehav. Rev..

[87] Kang S.Y., Kim Y.J., Jang W., Son K.Y., Park H.S., Kim Y.S. (2021). Body mass index trajectories and the risk for Alzheimer’s disease among older adults. Sci. Rep..

[88] Gustafson D., Rothenberg E., Blennow K., Steen B., Skoog I. (2003). An 18-year follow-up of overweight and risk of Alzheimer disease. Arch. Intern. Med..

[89] Govindugari V.L., Golla S., Reddy S.D.M., Chunduri A., Nunna L.S.V., Madasu J., Shamshabad V., Bandela M., Suryadevara V. (2023). Thwarting Alzheimer’s Disease through Healthy Lifestyle Habits: Hope for the Future. Neurol. Int..

[90] Kiliaan A.J., Arnoldussen I.A., Gustafson D.R. (2014). Adipokines: A link between obesity and dementia?. Lancet Neurol..

[91] Pasinetti G.M., Eberstein J.A. (2008). Metabolic syndrome and the role of dietary lifestyles in Alzheimer’s disease. J. Neurochem..

[92] Morris M.C. (2016). Nutrition and risk of dementia: Overview and methodological issues. Ann. N. Y. Acad. Sci..

[93] Colizzi C. (2019). The protective effects of polyphenols on Alzheimer’s disease: A systematic review. Alzheimers Dement..

[94] Wang J., Ho L., Zhao Z., Seror I., Humala N., Dickstein D.L., Thiyagarajan M., Percival S.S., Talcott S.T., Pasinetti G.M. (2006). Moderate consumption of Cabernet Sauvignon attenuates Abeta neuropathology in a mouse model of Alzheimer’s disease. FASEB J..

[95] Dhana K., Evans D.A., Rajan K.B., Bennett D.A., Morris M.C. (2020). Healthy lifestyle and the risk of Alzheimer dementia: Findings from 2 longitudinal studies. Neurology.

[96] Dhana K., Franco O.H., Ritz E.M., Ford C.N., Desai P., Krueger K.R., Holland T.M., Dhana A., Liu X., Aggarwal N.T. (2022). Healthy lifestyle and life expectancy with and without Alzheimer’s dementia: Population based cohort study. BMJ.

[97] Toro C.A., Zhang L., Cao J., Cai D. (2019). Sex differences in Alzheimer’s disease: Understanding the molecular impact. Brain Res..

[98] Barha C.K., Falck R.S., Skou S.T., Liu-Ambrose T. (2021). Personalising exercise recommendations for healthy cognition and mobility in aging: Time to address sex and gender (Part 1). Br. J. Sports Med..

[99] Kumar M., Srivastava S., Muhammad T. (2022). Relationship between physical activity and cognitive functioning among older Indian adults. Sci. Rep..

[100] Barha C.K., Davis J.C., Falck R.S., Nagamatsu L.S., Liu-Ambrose T. (2017). Sex differences in exercise efficacy to improve cognition: A systematic review and meta-analysis of randomized controlled trials in older humans. Front. Neuroendocrinol..

[101] Barha C.K., Starkey S.Y., Hsiung G.Y.R., Tam R., Liu-Ambrose T. (2023). Aerobic exercise improves executive functions in females, but not males, without the BDNF Val66Met polymorphism. Biol. Sex Differ..

[102] Gajewski P.D., Falkenstein M. (2015). Lifelong physical activity and executive functions in older age assessed by memory based task switching. Neuropsychologia.

[103] Benini R., Nunes P.R.P., Orsatti C.L., Portari G.V., Orsatti F.L. (2015). Influence of sex on cytokines, heat shock protein and oxidative stress markers in response to an acute total body resistance exercise protocol. J. Exerc. Sci. Fit..

[104] Safdar B., Jarman A.F., Madsen T.E., DeLamielleure L.E., Zhou B., Axtell R., Geirsson A., Mangi A.A. (2025). Sex Differences in Response to a 12-Week Resistance Training Exercise Intervention After Cardiac Surgery: A Proof-of-Concept Intervention Trial. Clin. Ther..

[105] Varma V.R., Chuang Y.F., Harris G.C., Tan E.J., Carlson M.C. (2015). Low-intensity daily walking activity is associated with hippocampal volume in older adults. Hippocampus.

[106] Moschny A., Platen P., Klaassen-Mielke R., Trampisch U., Hinrichs T. (2011). Physical activity patterns in older men and women in Germany: A cross-sectional study. BMC Public Health.

[107] Keyhani D., Tartibian B., Dabiri A., Teixeira A.M.B. (2020). Effect of High-Intensity Interval Training Versus Moderate-Intensity Aerobic Continuous Training on Galectin-3 Gene Expression in Postmenopausal Women: A Randomized Controlled Trial. J. Aging Phys. Act..

[108] Barha C.K., Best J.R., Rosano C., Yaffe K., Catov J.M., Liu-Ambrose T. (2020). Sex-Specific Relationship Between Long-Term Maintenance of Physical Activity and Cognition in the Health ABC Study: Potential Role of Hippocampal and Dorsolateral Prefrontal Cortex Volume. J. Gerontol. A Biol. Sci. Med. Sci..

[109] van Uffelen J.G., Chinapaw M.J., van Mechelen W., Hopman-Rock M. (2008). Walking or vitamin B for cognition in older adults with mild cognitive impairment? A randomised controlled trial. Br. J. Sports Med..

[110] Barha C.K., Hsu C.L., Ten Brinke L., Liu-Ambrose T. (2019). Biological Sex: A Potential Moderator of Physical Activity Efficacy on Brain Health. Front. Aging Neurosci..

[111] Fernández-Rodríguez R., Martínez-Vizcaíno V., Reina-Gutiérrez S., Bizzozero-Peroni B., Torres-Costoso A., Rodríguez-Gutiérrez E., Díaz-Goñi V., Cadenas-Sánchez C. (2024). Sex Differences in Effects of Exercise on Physical Function in Aging: A Systematic Review with Meta-Analysis. World J. Mens. Health.

[112] Barha C.K., Falck R.S., Davis J.C., Nagamatsu L.S., Liu-Ambrose T. (2017). Sex differences in aerobic exercise efficacy to improve cognition: A systematic review and meta-analysis of studies in older rodents. Front. Neuroendocrinol..

[113] White Z., Terrill J., White R.B., McMahon C., Sheard P., Grounds M.D., Shavlakadze T. (2016). Voluntary resistance wheel exercise from mid-life prevents sarcopenia and increases markers of mitochondrial function and autophagy in muscles of old male and female C57BL/6J mice. Skelet. Muscle.

[114] Barha C.K., Liu-Ambrose T. (2020). Sex differences in exercise efficacy: Is midlife a critical window for promoting healthy cognitive aging?. FASEB J..

[115] Barha C.K., Hsiung G.R., Best J.R., Davis J.C., Eng J.J., Jacova C., Lee P.E., Munkacsy M., Cheung W., Liu-Ambrose T. (2017). Sex Difference in Aerobic Exercise Efficacy to Improve Cognition in Older Adults with Vascular Cognitive Impairment: Secondary Analysis of a Randomized Controlled Trial. J. Alzheimers Dis..

[116] Baker L.D., Frank L.L., Foster-Schubert K., Green P.S., Wilkinson C.W., McTiernan A., Plymate S.R., Fishel M.A., Watson G.S., Cholerton B.A. (2010). Effects of aerobic exercise on mild cognitive impairment: A controlled trial. Arch. Neurol..

[117] Stojanovic M., Babulal G.M., Head D. (2023). Determinants of physical activity engagement in older adults. J. Behav. Med..

[118] García-Mesa Y., López-Ramos J.C., Giménez-Llort L., Revilla S., Guerra R., Gruart A., Laferla F.M., Cristòfol R., Delgado-García J.M., Sanfeliu C. (2011). Physical exercise protects against Alzheimer’s disease in 3xTg-AD mice. J. Alzheimers Dis..

[119] Naghibi S., Shariatzadeh Joneydi M., Barzegari A., Davoodabadi A., Ebrahimi A., Eghdami E., Fahimpour N., Ghorbani M., Mohammadikia E., Rostami M. (2021). Treadmill exercise sex-dependently alters susceptibility to depression-like behaviour, cytokines and BDNF in the hippocampus and prefrontal cortex of rats with sporadic Alzheimer-like disease. Physiol. Behav..

[120] Cortes C.J., De Miguel Z. (2022). Precision Exercise Medicine: Sex Specific Differences in Immune and CNS Responses to Physical Activity. Brain Plast..

[121] Zhou C.N., Chao F.L., Zhang Y., Jiang L., Zhang L., Luo Y.M., Xiao Q., Chen L.M., Tang Y. (2018). Sex Differences in the White Matter and Myelinated Fibers of APP/PS1 Mice and the Effects of Running Exercise on the Sex Differences of AD Mice. Front. Aging Neurosci..

[122] Kohman R.A., Bhattacharya T.K., Wojcik E., Rhodes J.S. (2013). Exercise reduces activation of microglia isolated from hippocampus and brain of aged mice. J. Neuroinflammation.

[123] Bretland K.A., Lin L., Bretland K.M., Smith M.A., Fleming S.M., Dengler-Crish C.M. (2021). Irisin treatment lowers levels of phosphorylated tau in the hippocampus of pre-symptomatic female but not male htau mice. Neuropathol. Appl. Neurobiol..

[124] Short A.K., Bui V., Zbukvic I.C., Hannan A.J., Pang T.Y., Kim J.H. (2022). Sex-dependent effects of chronic exercise on cognitive flexibility but not hippocampal Bdnf in aging mice. Neuronal Signal.

[125] Herring A., Donath A., Yarmolenko M., Uslar E., Conzen C., Kanakis D., Bosma C., Worm K., Paulus W., Keyvani K. (2012). Exercise during pregnancy mitigates Alzheimer-like pathology in mouse offspring. FASEB J..

[126] García-Mesa Y., Pareja-Galeano H., Bonet-Costa V., Revilla S., Gómez-Cabrera M.C., Gambini J., Giménez-Llort L., Cristòfol R., Viña J., Sanfeliu C. (2014). Physical exercise neuroprotects ovariectomized 3xTg-AD mice through BDNF mechanisms. Psychoneuroendocrinology.

[127] Chelucci E., Scarfò G., Piccarducci R., Rizza A., Fusi J., Epifani F., Carpi S., Polini B., Betti L., Costa B. (2024). Sex Differences in Blood Accumulation of Neurodegenerative-Related Proteins and Antioxidant Responses to Regular Physical Exercise. J. Mol. Neurosci..

[128] Giménez-Llort L., García Y., Buccieri K., Revilla S., Suñol C., Cristofol R., Sanfeliu C. (2010). Gender-Specific Neuroimmunoendocrine Response to Treadmill Exercise in 3xTg-AD Mice. Int. J. Alzheimers Dis..

[129] Chaudhari K., Wong J.M., Vann P.H., Como T., O’Bryant S.E., Sumien N. (2020). ApoE Genotype-Dependent Response to Antioxidant and Exercise Interventions on Brain Function. Antioxidants.

